# Identifying direct contacts between protein complex subunits from their conditional dependence in proteomics datasets

**DOI:** 10.1371/journal.pcbi.1005625

**Published:** 2017-10-12

**Authors:** Kevin Drew, Christian L. Müller, Richard Bonneau, Edward M. Marcotte

**Affiliations:** 1 Center for Systems and Synthetic Biology, Department of Molecular Biosciences, University of Texas at Austin, Austin, TX, United States of America; 2 Flatiron Institute, Center for Computational Biology, Simons Foundation, New York, NY, United States of America; 3 New York University Center for Genomics and Systems Biology, New York University, New York, NY, United States of America; University of California, San Diego, UNITED STATES

## Abstract

Determining the three dimensional arrangement of proteins in a complex is highly beneficial for uncovering mechanistic function and interpreting genetic variation in coding genes comprising protein complexes. There are several methods for determining co-complex interactions between proteins, among them co-fractionation / mass spectrometry (CF-MS), but it remains difficult to identify directly contacting subunits within a multi-protein complex. Correlation analysis of CF-MS profiles shows promise in detecting protein complexes as a whole but is limited in its ability to infer direct physical contacts among proteins in sub-complexes. To identify direct protein-protein contacts within human protein complexes we learn a sparse conditional dependency graph from approximately 3,000 CF-MS experiments on human cell lines. We show substantial performance gains in estimating direct interactions compared to correlation analysis on a benchmark of large protein complexes with solved three-dimensional structures. We demonstrate the method’s value in determining the three dimensional arrangement of proteins by making predictions for complexes without known structure (the exocyst and tRNA multi-synthetase complex) and by establishing evidence for the structural position of a recently discovered component of the core human EKC/KEOPS complex, GON7/C14ORF142, providing a more complete 3D model of the complex. Direct contact prediction provides easily calculable additional structural information for large-scale protein complex mapping studies and should be broadly applicable across organisms as more CF-MS datasets become available.

## Author summary

Proteins physically associate into complexes in order to carry out the essential functions of life. Knowing how proteins are physically arranged three dimensionally in these complexes provides clues towards how they work. In principle, the associations between proteins in large-scale proteomics datasets should often reflect direct physical contacts between proteins in each complex. Here, we describe a statistical method to discover which subunits within complexes directly contact each other based on their co-purification behavior in published co-fractionation mass spectrometry datasets. Within our predictions, we recover many known protein-protein contacts, serving to validate our method, as well as unknown contacts that can inform future studies of these complexes. Specifically, we observe confident contacts between subunits within the exocyst and tRNA multi-synthetase complexes, two complexes that have incomplete structural information. Using our method, we further provide structural information for a previously missing subunit of the EKC/KEOPS complex. We anticipate that this method and the associated predictions will help to better inform our understanding of the functions and structures of diverse protein complexes.

This is a *PLOS Computational Biology* Methods paper.

## Introduction

Many proteins assemble into large macromolecular complexes with essential cellular functions. The three dimensional arrangement of proteins in a complex is vital to the complex’s function and knowledge of this arrangement would be highly valuable in understanding the mechanism of function. Conserved protein complexes are estimated to number in the thousands but the vast majority of these are structurally elusive by traditional structural biology techniques. Advances in proteomics technologies have allowed for the high throughput identification of protein complexes across the tree of life including large-scale affinity purification mass spectrometry (AP-MS) datasets [1–3] as well as high-throughput co-fractionation mass spectrometry (CF-MS) datasets comprising thousands of experiments across human, metazoan and prokaryotes [4–7].

In the CF-MS approach, cellular lysate is biochemically fractionated by multiple, non-denaturing chromatographic methods and then complexes are inferred bioinformatically in a machine-learning framework using correlations of the resulting protein elution profiles as a prominent feature. Although this approach has primarily been used to identify component subunits of complexes, we previously observed that the correlation structure of the protein elution profiles also revealed structural information about the complexes [6]. This allowed for the identification of sub-complexes, which were accurate when compared to known structural models and when compared to known functions. However, correlation did not consistently reveal the directly bound protein pairs that other experiments such as yeast two-hybrid [8, 9] and chemical crosslinking [10–14] can reveal across large portions of the proteome. Other computational approaches have been proposed to identify direct contacts by analyzing co-occurrence of proteins in mass spectrometry experiments but they have only been applied to AP-MS datasets [15].

Protein sub-complexes are valuable in understanding the three dimensional arrangement of proteins in a complex but correlation often convolutes specific physical interactions between proteins with indirect interactions and non-physical relationships. Removal of these spurious interactions from the correlation network is crucial to identifying which specific proteins directly contact each other. A classical statistical approach to remove such interactions can be achieved with graphical models [16]. Graphical models represent the conditional dependence structure of a set of random variables as a graph. Unfortunately, classical statistical methods to estimate graphical models fail in scenarios where the number of variables (e.g., proteins) greatly exceeds the number of samples, such as the case with co-fractionation profiles. However, recent advances in the field of statistical analysis, specifically on the topic of sparse high-dimensional statistical inference, have led to new methods for addressing these underdetermined problems (see, e.g. [17] and references therein). In biology, these methods enabled a number of successful applications of graphical modeling, such as estimating interactions between genes from high-throughput expression profiles [18], predicting contacts between amino acid residues from multiple sequence alignments [19], and inferring associations of microbes from environmental sequencing data [20], respectively.

**Fig 1:**
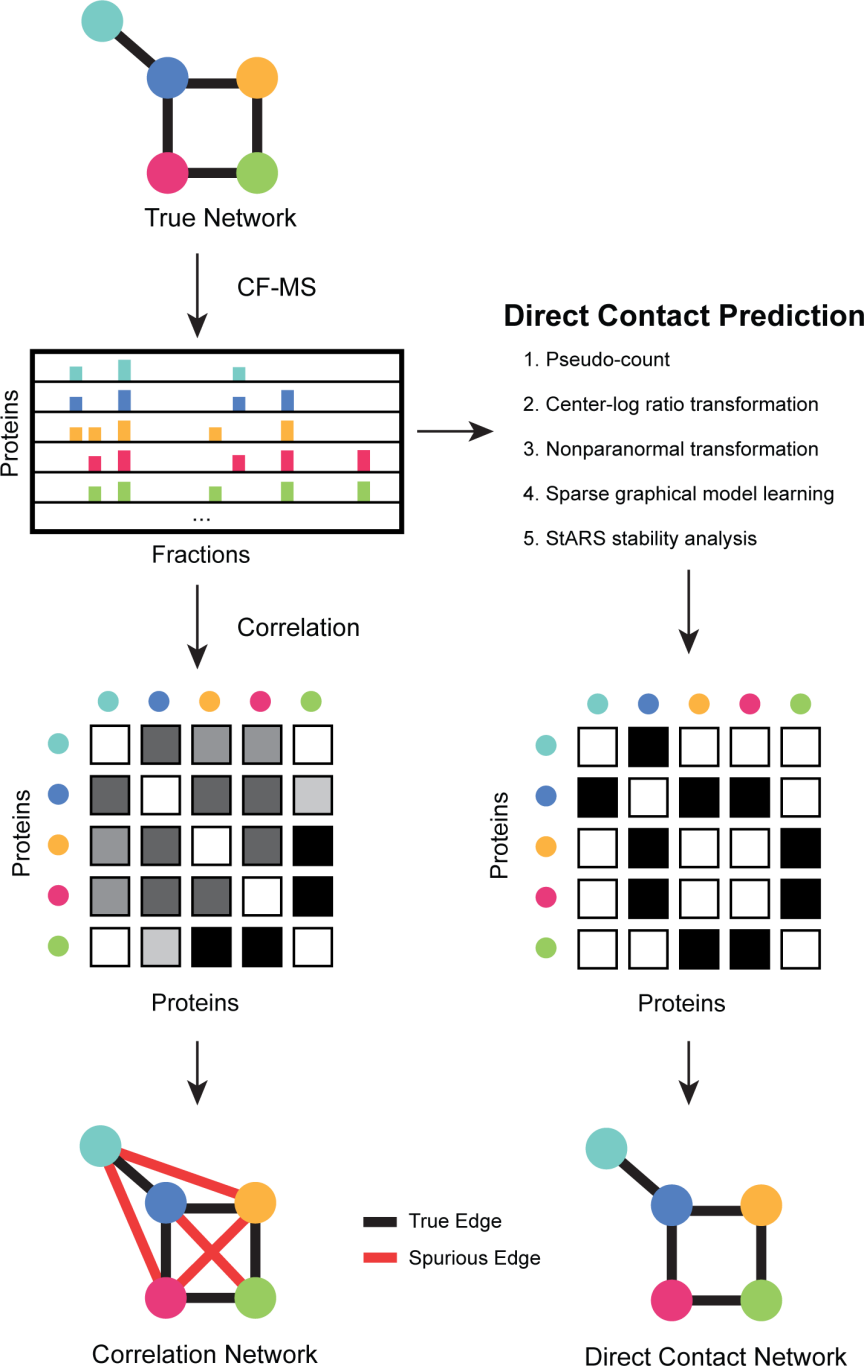
Overview of direct contact prediction between protein complex subunits. Co-fractionation / mass spectrometry (CF-MS) aims to repeatedly separate mixtures of native protein complexes (True Network) by non-denaturing chromatography. Protein elution profiles are generated by mass spectrometry identification of proteins across all chromatography fractions collected. Correlation between proteins’ elution profiles (**left side**) performs well for identifying the subunit composition of complexes [4, 6, 7], but suffers from indirect associations among proteins that inhibit its ability to identify directly contacting subunits within each complex. We predict direct contacts (**right side**) by effectively inverting the correlation matrix to discriminate between conditionally dependent and conditionally independent associations, which correspond to direct and indirect protein interactions respectively. Specifically, we incorporate pseudo-counts, scale and transform the correlation matrix, use a sparse graphical model learning framework to compute conditionally dependent partial correlations, followed by StARS stability analysis [29] to re-score the resulting conditional dependency matrix such that each entry corresponds to the frequency with which it is supported by subsample trials. We retain non-zero scores between subunits within each pre-defined human protein complex [32] as our prediction of direct contacts.

Here, we apply a graphical model to identify direct protein interactions (Fig 1) from one of the largest proteomic interaction datasets to date consisting of approx. 3,000 published human CF-MS experiments [6]. We make the assumption that conditional dependence is a proxy for direct protein interactions, which is consistent with the biochemical chromatography methods used in CF-MS experiments due to their separation of native complexes and sub-complexes. We evaluated the performance of our predictions in a precision-recall framework on a benchmark of large protein complexes with known molecular structures and observe substantial improvement over correlation alone. We also observe that the ranking of the learned conditional dependencies is insensitive to particular choices of the regularization parameter *λ* which balances model complexity and model fit. We additionally characterize our method’s performance finding better predictions for well-observed complexes and validate our predictions with a whole cell lysate crosslinking dataset where we observe enriched overlap. We therefore believe, in principle, these measures of conditional dependence could also be applied to additional proteomic datasets such as AP-MS as well as used in conjunction with other features of direct protein-protein contacts in supervised machine learning frameworks to further improve predictive performance.

We highlight predictions made for the 26S proteasome complex and demonstrate agreement with the true set of contacts. We show new predictions for complexes without known structures, specifically the exocyst and tRNA multi-synthetase complex, to illustrate the utility of our approach. Finally, in our predicted set of directly contacting proteins we show support for direct contact of a recently identified component of the human EKC/KEOPS complex. Our results suggest that our predicted direct protein interaction edges will be a valuable constraint that can be used in structurally modeling the thousands of stable protein complexes in the human proteome inaccessible to current structure determination techniques, as we demonstrate with an improved 3D model of the EKC/KEOPS complex.

## Methods

### Calculation of conditionally dependent protein-protein interactions

In order to identify direct physical interactions between proteins, we first organized a large, published dataset of human CF-MS experiments [6]. CF-MS experiments consist of two steps, the first being to biochemically separate native protein complexes and sub-complexes along a specified gradient (e.g., hydrodynamic radius, charge, etc.) using non-denaturing separation techniques that preserve intact complexes. The second step is to identify and quantify the proteins that elute at each time point, providing a characteristic elution profile for each protein observed. The aim of our approach is to use these elution profiles to reconstruct the physical interaction network of the proteins identified, and specifically find which proteins directly contact each other within complexes.

The dataset comprises *d* = 15,964 protein elution profiles each consisting of a vector of *n* = 2,989 protein abundance values. Each protein abundance value is derived from 28 fractionation experiments using multiple, distinct biochemical separation techniques, including ion exchange chromatography, isoelectric focusing and sucrose gradients, analyzing native protein extracts isolated from HeLa cells (17 experiments), HEK293 cells (8), glioma stem cells (2) and neural stem cells (1). Fractionation experiments consist of a series of collected fractions along a biochemical gradient of the applied chromatography method. The number of fractions ranges between ~10 to ~200 per experiment depending on the method. Each fraction of extract is then subjected to proteomic analysis using mass spectrometry producing observed protein abundances. We use the pipeline described in Wan *et al*. 2015 [6], where the proteomic consensus identification tool, MSBlender [21] is used to identify proteins from mass spectra. For peptide identifications, we use a false discovery rate of < 1%. Missing values that arise when a protein is not identified in a given fraction are set to 0.0. This diverse set of experimental conditions allows for the analysis of a large fraction of the proteome and thorough separation of endogenous complexes. We denote the resulting CF-MS data matrix by $X\in R_{0}^{d\times n}$. Each column *X*_*i*_, *i* = 1,…,*n* represents relative protein abundance data (compositions) and is normalized to sum up to 1.

We next introduce a sparse graphical model learning framework to infer direct (physical) protein interactions from CF-MS data from the covariation pattern of the protein abundances. Here, the nodes of the graph represent proteins and the edges approximate direct protein contacts. We first note that components of the compositions *X*_*i*_ are not independent due to the unit sum constraint. Thus, higher order statistics, such as covariance matrices of compositional data exhibit negative bias due to closure. To alleviate this shortcoming we borrow a transformation technique from compositional data analysis [22], the so-called centered log-ratio (CLR) transformation. The CLR transformation is defined as $\mathrm{CLR}(X_{i})=\log\frac{X_{i}}{g(X_{i})}$, where *g*(*X*_*i*_) denotes the geometric mean. This transformation is particularly useful, as it is symmetric and isometric with respect to the original composition. The CLR maps compositional data from the *d*-dimensional simplex to a (*d* − 1)-hyperplane in *d*-dimensional Euclidean space. A pseudo-count of 1 is added to all entries in *X* to ensure applicability of the transformation. We denote the corresponding covariance matrix by Γ = cov(CLR(*X*)).

Recent work [23] has shown that, in the sparse high-dimensional setting and under certain technical conditions, the covariance matrix Γ is a good estimator for the covariance matrix Σ ∈ ℝ^*d*×*d*^ of the unknown absolute abundances. This observation is the basis for the proposed graphical model inference framework. Following [20, 24], we propose to learn a sparse undirected graph $G\in R^{d\times d}$ representing node-node interactions *via* the following minimization problem:

$$\hat{G}(\lambda )={\mathrm{argmin}}_{G\in R^{d\times d},G_{jj}=0}\frac{1}{2}\mathrm{tr}(G^{⊺}\Gamma G)-\mathrm{tr}(G^{⊺}\Gamma )+\lambda {\left\|G\right\|}_{1}$$

for all *j* = 1,…,*d* where tr denotes the trace operator, ‖∙‖_1_ denotes the element-wise L1 norm, and *λ* > 0 is the regularization parameter. Each of the *d* subproblems is equivalent to fitting a linear regression model with L1 penalization (Lasso) [25] to each protein profile, using the other profiles as predictors. To relax any distributional dependencies of the regression, we also apply a non-paranormal (copula) transform to the data before the linear regression step [12]. To symmetrize the graph, derived from the described node-wise regression (or neighborhood selection) algorithm, the OR rule is applied across all node neighborhoods, *i*.*e*., an edge in the protein-protein graph is present if either node *i* is associated with node *j* or vice versa. It has been shown in [24] that, under certain conditions, the non-zero entries ${\hat{G}}_{ij}\neq 0$ of this symmetrized adjacency matrix are asymptotically identical to the non-zero elements Θ_*ij*_ of the inverse covariance (or precision) matrix Θ = Σ^−1^. This allows a clear statistical interpretation of the edges in terms of partial correlation coefficients among the nodes [26]. Thus, the procedure is able to remove transitive correlations among nodes by approximately learning the full conditional dependence among all nodes.

### Model selection and interaction ranking

One of the key challenges in learning a sparse graphical model from data is the selection of the regularization parameter *λ* > 0. In the unsupervised setting, several methods have been proposed, including cross validation and information criteria [27, 28]. One state-of-the-art model selection scheme is the Stability Approach to Regularization Selection (StARS) [29]. StARS selects the minimum amount of regularization that results in a graph that is sparse and comprises a stable edge set under random subsampling of the data at a prescribed stability level 1 − *β* [30, 31]. StARS typically selects *N* = 20 sub-samples of size $b(n)=\lfloor 10\sqrt{n}\rfloor$ and learns a graphical model from each subsample across the entire *λ*-path (here, 30 values of *λ* are chosen between 0 and *λ*_max_). StARS records for each edge in $\hat{G}(\lambda )$ the empirical frequency of edge presence *P*_*ij*_ across the entire *λ*-path, stored in a list of matrices P(*λ*) ∈ [0,1]^*d*×*d*^. Standard StARS selects *λ* where the normalized sum of variances of the *P*_*ij*_ in the corresponding P(*λ*) drops below *β* = 0.1. It has been shown in [31] that this selection can lead to sub-optimal regularization selection. In the present application, we thus opted for an alternative semi-supervised selection procedure. For all positive edges in the interaction graph, we interpreted the edge frequencies as (protein) contact probabilities and ranked edges in order of decreasing contact probability. We compared these ranked predictions to a benchmark of physically interacting proteins determined from multi-protein complexes with known three-dimensional structures and selected the *λ* that maximized the area under the precision recall (AUPR) curve. The selected *λ* corresponds to a more conservative StARS variability threshold of *β* = 0.005. We also note that, in our application, our introduced edge ranking based on the edge stability was insensitive to the precise selection of *λ*.

Finally, we filtered our reported direct contact predictions by protein interactions that are present in 896 complexes larger than 4 subunits from the human protein complex map, hu.MAP [32]. This step was to ensure pairs of proteins are present in the same complex thereby increasing the likelihood of direct contact.

All computation was performed in R using the Hotelling package [33] for CLR transformation and the Huge [34] package for graphical modeling.

### Correlation analysis

For comparison purposes, correlation analysis was applied to each pair of protein co-elution profiles in the human CF-MS dataset. Profiles were first normalized by the total number of theoretical tryptic peptides for each protein and then a z-score was calculated for each value in the matrix relative to its corresponding fraction (*i*.*e*., column-wise standardization). Pearson correlation coefficients were then calculated for each pair of proteins.

### Assembly of a multi-protein complex structural benchmark

In order to evaluate the predictive performance of our direct contact prediction method we assembled a benchmark of 29 large non-redundant protein complexes with known structure (**S1 Table**). Due to the ease at which direct contacts can be predicted at random for small complexes, we restrict our benchmark to complexes having > 4 unique subunits. Note, subunits from certain complexes may not be sampled in our data or have ambiguous ortholog mapping. We process the reported biological assembly of each complex using the PISA tool [35], which calculates macromolecular interface surface area. All pairs of proteins within each complex with interfacial areas (Å^2^) > 0.0 were considered physically contacting and marked a true contact, comprising benchmark positive examples. Protein pairs with no interface area were considered not contacting, comprising benchmark negative examples. Note that protein pairs that spanned two complexes (e.g., protein 1 in complex 1 and protein 2 in complex 2) were not considered. For complexes whose structure was determined in an organism other than human, InParanoid [36] was used to identify human orthologs of the structurally solved subunit. If no human ortholog could be found for a given subunit, interactions involving that subunit were not considered. We split the benchmark into two sets, the first (10 complexes) to evaluate *λ* selection and performance and the second (19 complexes) to evaluate generality of the method. The complete protein pair benchmark is provided in **S2 Table**.

### Overlap enrichment of inter-protein crosslinking dataset

We evaluated the overlap of our direct contact predictions with a set of identified inter-protein crosslink interactions from Liu *et al*. [10]. Similar to the method described in [32] we collapsed all crosslink interactions to one interaction per pair of proteins. We first generate a random overlap distribution by selecting random pairs of proteins from the crosslinking dataset and calculate the overlap with the direct contact predictions for 1000 repeated trials. We then calculate a z-score for the overlap of the direct contact predictions and the reported crosslinking interactions with regards to random distribution. We repeat the process for determining the enrichment of complexes from hu.MAP and the crosslinking interactions.

### Structural modeling of the EKC/KEOPS complex

To construct a structural model of the human EKC/KEOPS complex, we built structural models of human EKC/KEOPS proteins based on available template structures in the Protein Data Bank (PDB) [37] and then aligned those models with existing co-complex structures. Specifically, we used HHPred [38] to build alignments of the query protein and PDB sequences and then used MODELLER [39] to build homology models. Homology models of human proteins were then structurally aligned to the homologous structures in yeast and archeal crystal structures [40–42] using DaliLite [43].

## Results and discussion

### Discovery of conditionally dependent protein interactions

**Fig 1** shows a workflow of our direct contact prediction framework. Native complexes represented by the true physical interaction network are biochemically fractionated and their proteins identified using mass spectrometry. In order to find pairwise relationships between proteins in a given CF-MS dataset, prior work has relied on correlation analysis, which effectively reconstructs the subunit composition of complexes (especially when used as features in a supervised machine learning framework, a case we do not consider here), but only partially indicates the direct binding relationships among those subunits [4, 6].

More specifically, using correlation to identify pairwise relationships results in a large fraction of indirect interactions. For example, consider proteins A, B and C, where A directly binds B, B directly binds C, but A does not directly bind C. In this scenario, a network based on correlation would produce a spurious edge between proteins A and C due to the indirect relationship mediated by protein B. To address this issue, the inverse covariance matrix can be calculated, which represents a network of undirected edges between conditionally dependent nodes. With respect to CF-MS data, the nodes represent proteins and the conditional dependence edges represent direct physical contacts.

The construction of this network has many theoretical solutions due to the limited number of samples and vast number of possible interactions, but methods are available to infer the inverse covariance matrix when the resulting network is expected to be sparse. Sparsity is a safe assumption with respect to protein interactions, as estimates of the total number of expected human protein-protein interactions range between 150k – 650k, orders of magnitude less than the roughly 200–300 million possible interactions [44–46].

As described in detail in the Methods, we analyzed a dataset of approx. 3,000 co-fractionation / mass spectrometry experiments [4, 6], and restrict direct contact predictions to known co-complex interactions. Specifically, we use complexes with structures in the PDB for evaluation and a set of 896 protein complexes larger than 4 unique subunits derived from >9000 published mass spectrometry proteomics experiments [1, 3, 4, 6] in hu.MAP [32], for all other predictions. In all, we identified 2,434 potential interactions (**S3 Table**).

### Conditionally dependent interactions are more likely to represent directly contacting subunits within complexes

**Fig 2:**
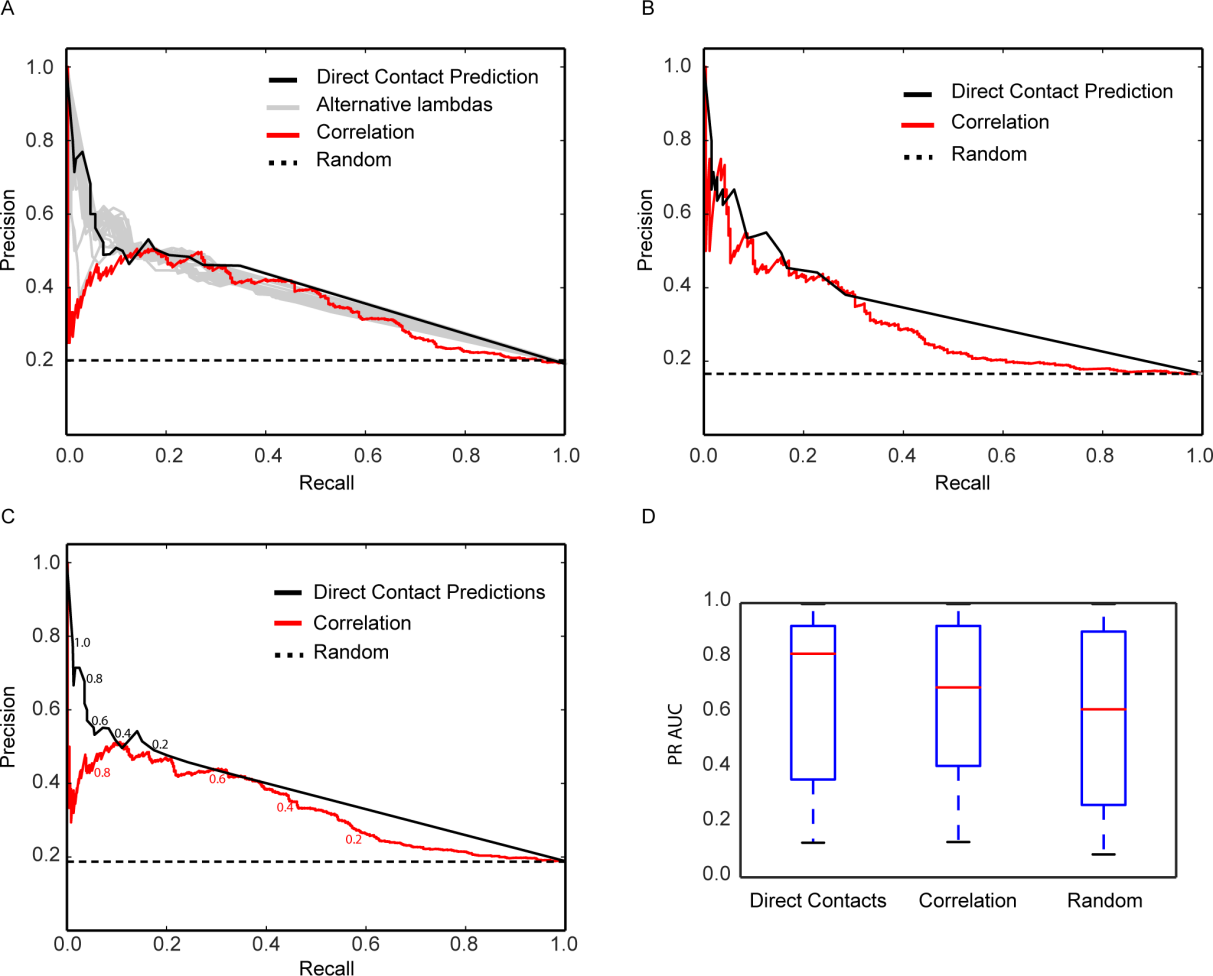
Scoring protein interactions by their conditional dependence accurately recovers direct protein-protein contacts within multi-subunit complexes. **A.** We compared the value of the pairwise Pearson correlation coefficients between protein elution profiles (red curve) versus the derived conditionally dependent interactions (*i*.*e*., direct contact predictions) (black curve) for their ability to recapitulate true protein contacts in 10 complexes with known 3D structures. High-scoring conditionally dependent interactions were strongly enriched for true contacts, unlike the most highly correlated protein elution profiles. Additionally, we plot precision recall curves for predictions made with alternative *λ* choices (gray curves) and observe improved performance over correlation alone suggesting performance is robust to the selection of this parameter. The random line (dashed) represents the theoretical baseline for all true positives (TP) divided by the total number of possible subunit pairs (TP:335 / Total:1583) **B.** Evaluation of conditionally dependent interactions on an additional 19 non-redundant complexes showing consistent performance on a leave out set. Random = (TP:261 / Total:1575). **C.** Evaluation on combined 29 complexes used in **A** and **B**. Direct contact probability thresholds and correlation coefficient thresholds are marked in black and red text, respectively. Random = (TP:596 / Total:3158). **D.** Distributions of area under the precision recall curve (PR AUC) for the individual 29 complexes showing large variance across complexes but showing direct contacts outperforming correlation and random. Precision = TP/(TP+FP); recall = TP/(TP+FN).

To evaluate whether our direct contact prediction method accurately identifies true interactions, we compared our predictions to a benchmark of physically interacting proteins determined from multi-protein complexes with known three-dimensional structures (**S1 Table**), as described in the Methods. **Fig 2A** plots the precision recall curve of our direct contact prediction method relative to the set of 10 complexes used to select *λ*. We observed high precision for the most confidently predicted contacts. This performance is in contrast to correlation analysis, also plotted in **Fig 2A**, which has limited accuracy for high correlation coefficients. Plotting precision-recall curves for the 29 alternative *λ* values considered during *λ* selection (**Fig 2A**, gray curves) confirmed that all predictions made with alternative *λ* values substantially outperformed correlation alone, demonstrating that this parameter was highly stable with regard to its selected value.

We further evaluated our direct contact predictions on an additional 19 complexes with known structure (**Fig 2B**) and observe consistent behavior of our method in terms of precision recall. Interestingly, while correlation performs poorly relative to our method including all *λ* values on the first set of complexes, it performs substantially better on the second benchmark almost equal to our method. The precision recall curve of the combined benchmark with both direct contact probability and correlation threshold markers can be found in **Fig 2C**.

We next asked if the ability to predict direct contacts was consistent across all complexes or if certain complexes performed better than others. We therefore calculated the area under the precision recall curve (PR AUC) for each individual complex and plotted its distribution in **Fig 2D**. For our direct contact predictions, we observe a large variance of PR AUC suggesting our method performs well for certain complexes and is limited for others. We still find, however, direct contact predictions outperform correlation analysis and random predictions.

### Direct contact predictions are made for well-observed complexes

**Fig 3:**
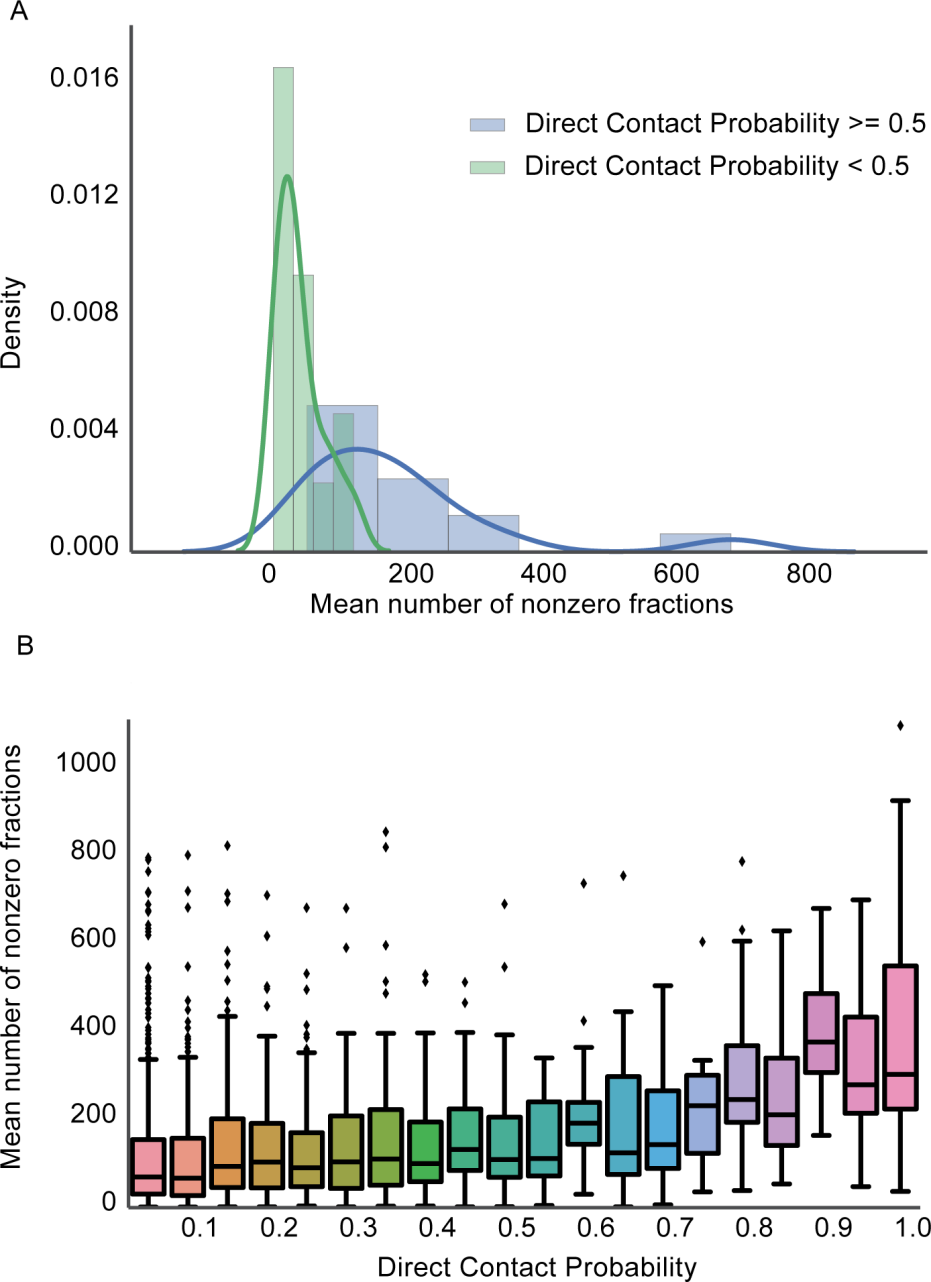
The direct contact prediction method makes high confident predictions for well-sampled complexes. **A.** Distribution of protein subunit sampling for complexes with a max direct contact probability > = 0.5 (blue) and complexes with a max probability < 0.5 (green). Sampling is measured for a complex by averaging the number of fractions for which each protein in the complex is observed (i.e. nonzero fractions). Our method performs better for well-sampled complexes and is limited for poorly sampled ones. **B.** Distribution of the mean number of nonzero fractions for pairs of proteins predicted by the direct contact method. Pairs of proteins with high probabilities are well sampled compared to those with lower probabilities.

To further understand what types of complexes for which our method is appropriate, we investigated how much of an impact the amount of experimental observation affected the degree to which high confident direct contact predictions were made. We first calculated the number of nonzero protein abundance measurements (i.e. count of fractions) for each observed protein and then computed the mean count for every complex in the structure benchmark. **Fig 3A** shows the distribution of the mean counts for complexes that had at least one prediction with a direct contact probability > = 0.5 and those complexes which did not. We observe a difference in the distributions suggesting that complexes that are well sampled in our dataset are more likely to have high confident predictions. It is important to note that several complexes in our benchmark are not well sampled and our method errs on the side of false negatives so as to limit making false predictions. We additionally plot all direct contact predictions in **Fig 3B** to better understand the relationship between the direct contact probability score and amount of experimental sampling. We see that pairs of proteins that have high confidence predictions are more likely to have been well sampled suggesting that repeated observations of the proteins across many experiments are important. This trend is likely due to our subsampling scoring procedure which is robust to spurious co-elutions from a single experiment.

**Fig 4:**
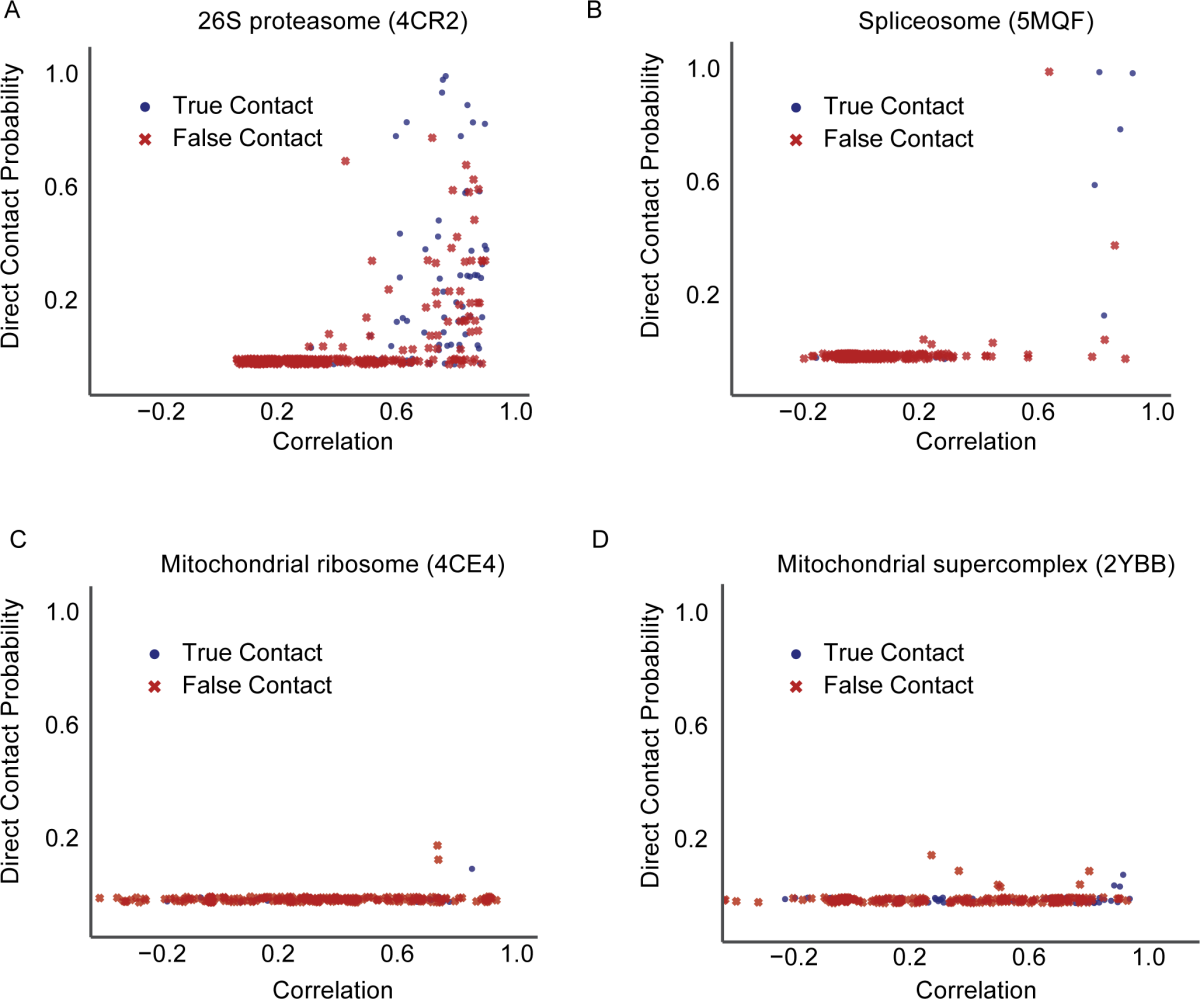
Relationship between direct contact probability and correlation for four example complexes with known structure. **A.** Direct contact predictions for the well-observed proteasome (pdbid: 4CR2) show good discrimination between true (blue circle) and false positives (red x) compared to that of correlation. **B**. Direct contact predictions for the moderately observed spliceosome complex (pdbid: 5MQF) shows good discrimination between true and false positives but with a limited number of total predictions. **C.** The direct contact method does not make high confident predictions for the mitochondrial ribosome (pdbid: 4CE4) due to its limited sampling while correlation makes many high ranking false positive predictions. **D.** Similar to **C**, the direct contact method does not make predictions for the mitochondrial super-complex (pdbid: 2YBB) due to its limited sampling while correlation makes several high confident true positive predictions.

**Fig 4** shows the relationship between correlation and direct contact probability for four examples of complexes in our structural benchmark spanning a range of well observed to poorly observed. Two of the complexes, the proteasome and spliceosome have high confidence predictions made by our method, while the other two, mitochondrial ribosome and mitochondrial super-complex have high-ranking correlation analysis predictions but lack high ranking predictions by our method. The proteasome (pdbid: 4CR2) is well observed with an average nonzero fraction count of ~356. **Fig 4A** shows our method makes many high confident true positive contact predictions for the proteasome (i.e. top 9/10 are correct) while protein pairs with high correlation coefficient have more of a mix of true positive and false positives. The spliceosome (pdbid: 5MQF, **Fig 4B**) is moderately observed in the dataset with an average nonzero fraction count of ~182 and still shows good relative discrimination between true and false positive contacts (i.e. top 5/10 are correct). Most of the co-fractionation experiments were focused on identifying soluble cytosolic complexes and therefore membrane bound complexes as well as complexes in subcellular compartments have limited coverage. For example, two mitochondrial complexes, the mitochondrial ribosome (pdbid: 4CE4, **Fig 4C**) and the mitochondrial super-complex (pdbid: 2YBB, **Fig 4D**) are identified in a limited number of fractions, on average ~42 and ~105 nonzero fractions respectively. Our method makes very few direct contact predictions for both complexes while correlation has a wide distribution of coefficients, many receiving high scores. Interestingly, high correlation coefficients for the mitochondrial ribosome have a high false positive rate (i.e. top 10 are all false positives) while the mitochondrial super-complex performs better with 7 out of the top 10 pairs being true positives. The poor performance on the mitochondrial ribosome by correlation analysis contributes to the substantial dip in performance seen in the precision recall curves (**Fig 2A**). These examples further demonstrate the ability of the direct contact prediction method to balance true and false positives and to accurately report contacts when sufficient data is available. As more CF-MS experimental datasets are published, we anticipate an improvement in the coverage of moderately to lowly observed complexes.

### Conditionally dependent interactions have high overlap with inter protein crosslinks

**Fig 5:**
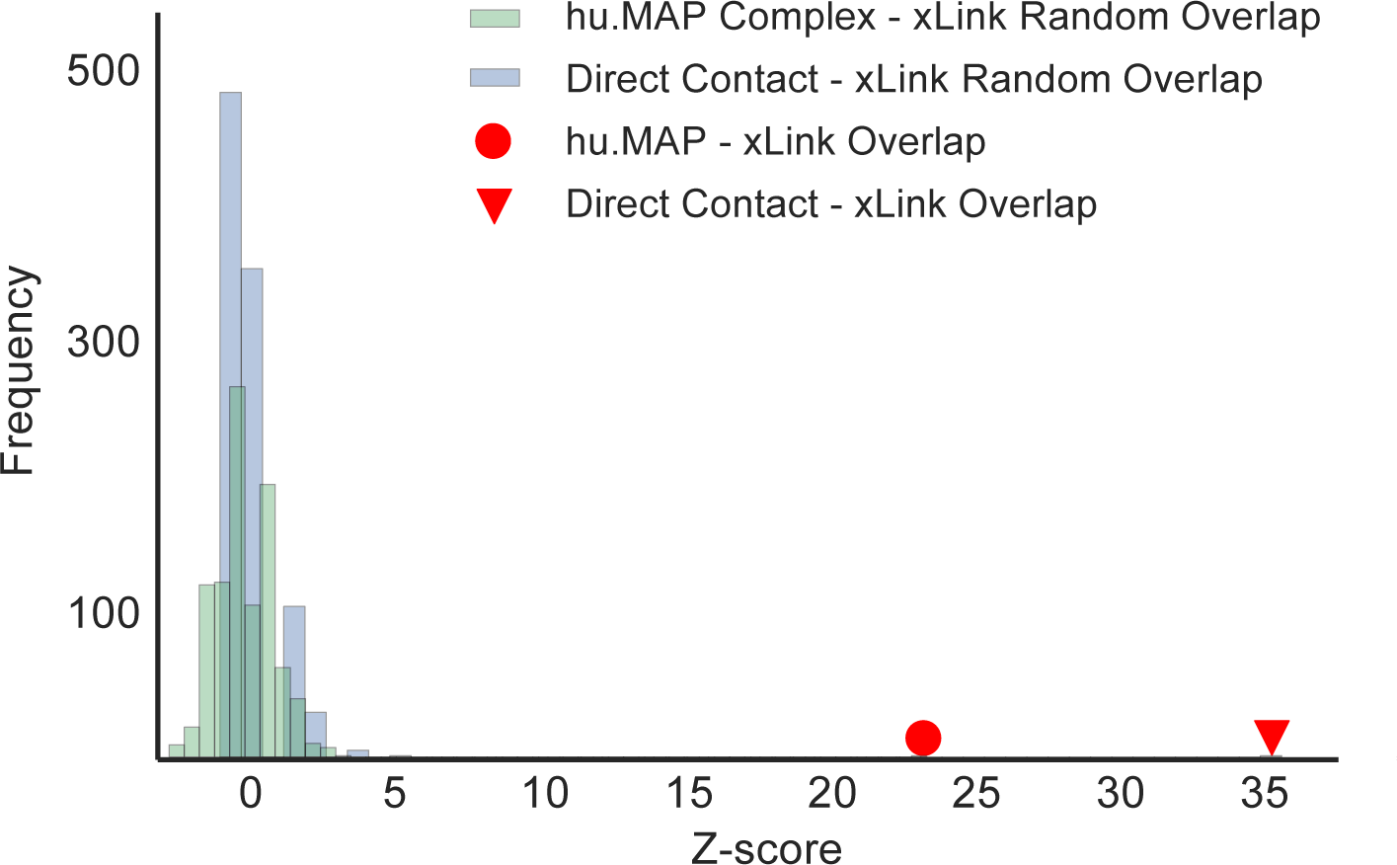
Direct contact predictions have highly enriched overlap with HeLa lysate crosslinking interactions. We report the enriched overlap of direct contact predictions and crosslinking interactions (z-score = 36, red triangle) relative to a distribution of random pairs of proteins in the crosslinking dataset (blue). Since we restrict our direct contact predictions to co-complex interactions within hu.MAP complexes, we additionally compare to the enriched overlap of co-complex edges and crosslinking interactions (z-score ~24, red circle). This shows direct contact predictions have a highly enriched overlap with crosslinking interactions above expected by co-complex edges alone.

To assess our direct contact predictions on an independent dataset different from protein structures, we compared to a human cell lysate mass spectrometry crosslinking dataset [10]. The maximum Cα-Cα distance between cross-linked residues for the DSSO cross-linker reagent used is 23.4 Å, making an identified cross-linked subunit pair a reasonable proxy for directly contacting proteins. Since our direct contact predictions are limited to co-complex subunits, we first compare the crosslinking dataset to the set of complexes with which we restricted our predictions. **Fig 5** shows that the overlap of complex edges and cross-linked subunits as well as the overlap of our conditionally dependent interactions and cross-linked subunits are both enriched compared to random pairs. Further, we see a much larger enrichment in our conditionally dependent interactions as opposed to complex edges demonstrating the direct contact predictions are highly enriched for physically close and contacting proteins pairs in human cell lysate.

### Evaluation of direct contacts within the 26S proteasome

**Fig 6:**
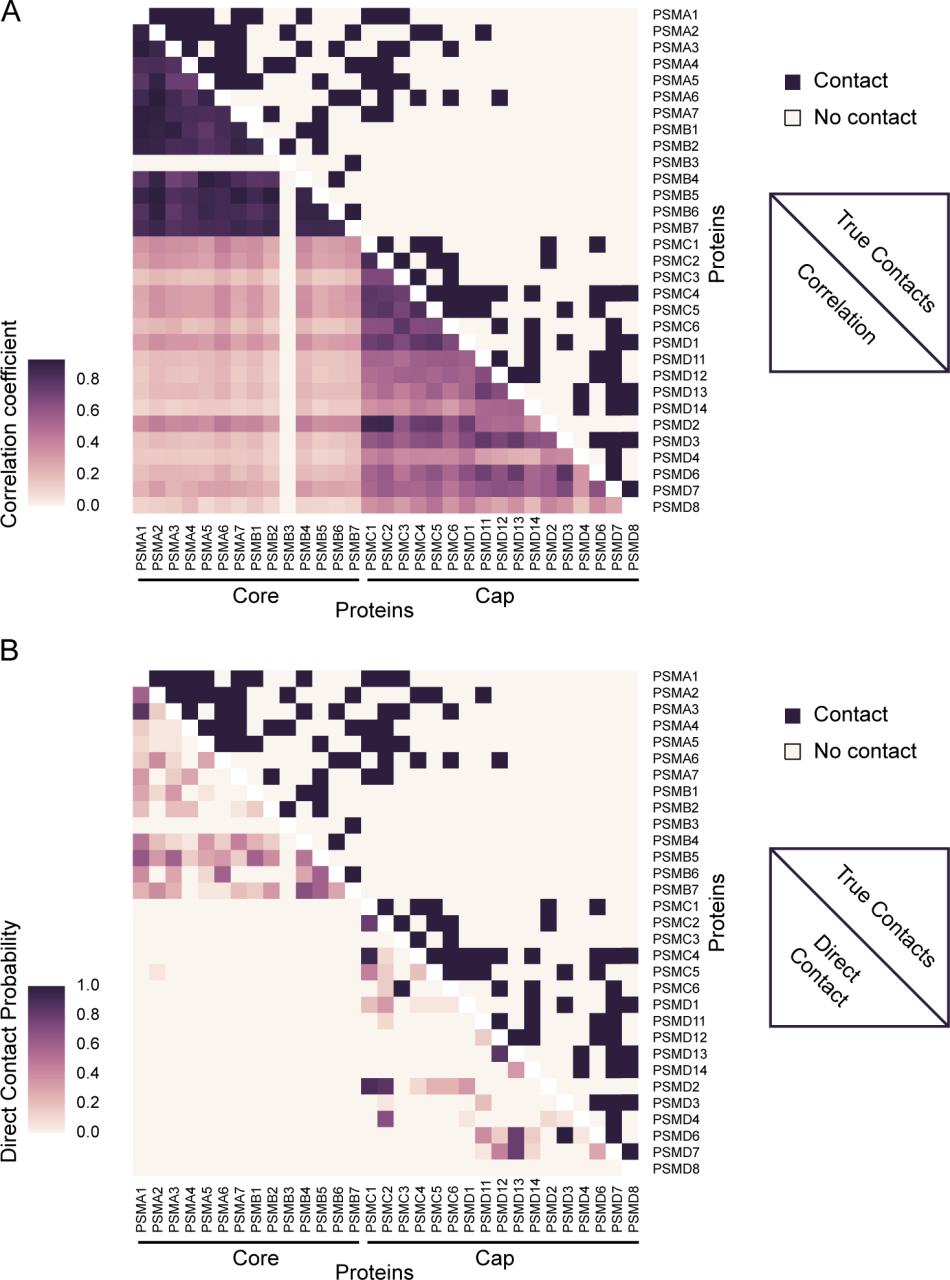
Prediction of direct contacts between subunits of the 26S proteasome. **A.** Matrix of true contacts (upper right, derived from PDB entry 4CR2 [47]) and correlation coefficients (lower left) for the 26S proteasome. Correlation identifies general sub-complex structure but fails to discriminate between direct and indirect interactions (6 out of top 10 predictions correct). **B.** Matrix of true contacts (upper right) and direct contact predictions (lower left) for the 26S proteasome. The direct contact method identifies many true contacts while strongly reducing the number of false positive predictions (9 out of top 10 predictions correct).

We next highlight our method’s ability to identify direct physical contacts among proteins by focusing on a specific protein complex with known structure. The 26S proteasome makes for a clear example of the utility of conditional dependency inference over correlation analysis due to the availability of known three-dimensional structures of this complex [47–50] and the presence of well-defined sub-complexes (e.g., the 20S core and 19S cap). **Fig 6A** shows the contacts from the known proteasome structure in the upper right portion of the matrix. Interactions are observed amongst the PSMA1 through PSMA7 subunits and PSMB1 through PSMB7 subunits, representing the core, as well as PSMC1 through PSMC6 and PSMD1 through PSMD14 subunits, representing the cap. Notably, not all subunits of the core contact each other, and there are relatively few contacts made between core and cap subunits. These known contacts can be compared with the case shown in the lower left portion of the matrix in **Fig 6A**, which plots correlation scores from fractionation profiles. While the correlation data exhibit a clear block structure with respect to the core and cap, they do not exhibit the more detailed structure observed in the true contact matrix.

The conditionally dependent interactions for these same data are plotted in the lower left portion of the matrix in **Fig 6B**, representing the method’s estimate of directly contacting subunits. In contrast to the full block structure exhibited by the raw correlations, the direct contact predictions capture finer details of the true contact matrix. Notably, many of the spurious indirect contacts predicted by the correlation matrix are successfully eliminated. For example, the core subunit PSMA6 does not directly contact PSMA1, PSMA7 or PSMB1-5, but does directly contact PSMA2-5 and PSMB6-7. This binding specificity is at least partly captured by the direct contact predictions, but is completely missed by the correlation analysis. Specifically, our method predicts no direct contacts between PSMA6 and PSMA7 or PSMB1-3 subunits, while correlation analysis produces high correlation coefficients for all core subunits. This example exposes the inability of correlation to identify specific direct physical contacts amongst indirect contacts and demonstrates the capacity to remove spurious contacts based on identification of conditional independence.

### Several high confidence false positive predictions are biologically meaningful

We looked further into cases were we predicted high confidence direct contacts that were labeled as incorrect based on structure data. We noticed an incorrect but high confidence direct contact prediction between two subunits of the spliceosome, SNRPD2 and SNRPD3 (direct contact prob = 1.0). The electron microscopy structure of the spliceosome (pdbid: 5MQF) shows these two subunits within ~17 Å of each other and between the two subunits is an RNA molecule. CF-MS is primarily a proteomics technique and does not observe other molecules such as RNA. We therefore expect to have a degree of error with respect to complexes with structural RNA present, as CF-MS will not show co-elution profiles that discriminate RNA—protein sub-assemblies. We do believe that when these data do become available, the direct contact prediction method is robust enough to identify direct contacts between RNA and protein molecules. Thus, in this case, the high confidence prediction points to a close biological relationship between the two subunits.

Additionally, we predict a high confidence direct contact (direct contact prob = 0.95) between two subunits of the eIF3 complex, specifically eIF3e and eIF3h. The C-termini of these subunits participate in an octameric helical bundle at the center of the complex but do not directly contact in the structure used for evaluation (pdbid 5A5T) [51]. In contrast, another structure of eIF3 (pdbid: 3J8B) [52] does have eIF3e and eIF3h directly contacting in the helical bundle. Both structures have limited resolution and are not considered atomic-models suggesting that our data can inform in this discrepancy between models.

### Prediction of direct contacts within human protein complexes with unknown structures

**Fig 7:**
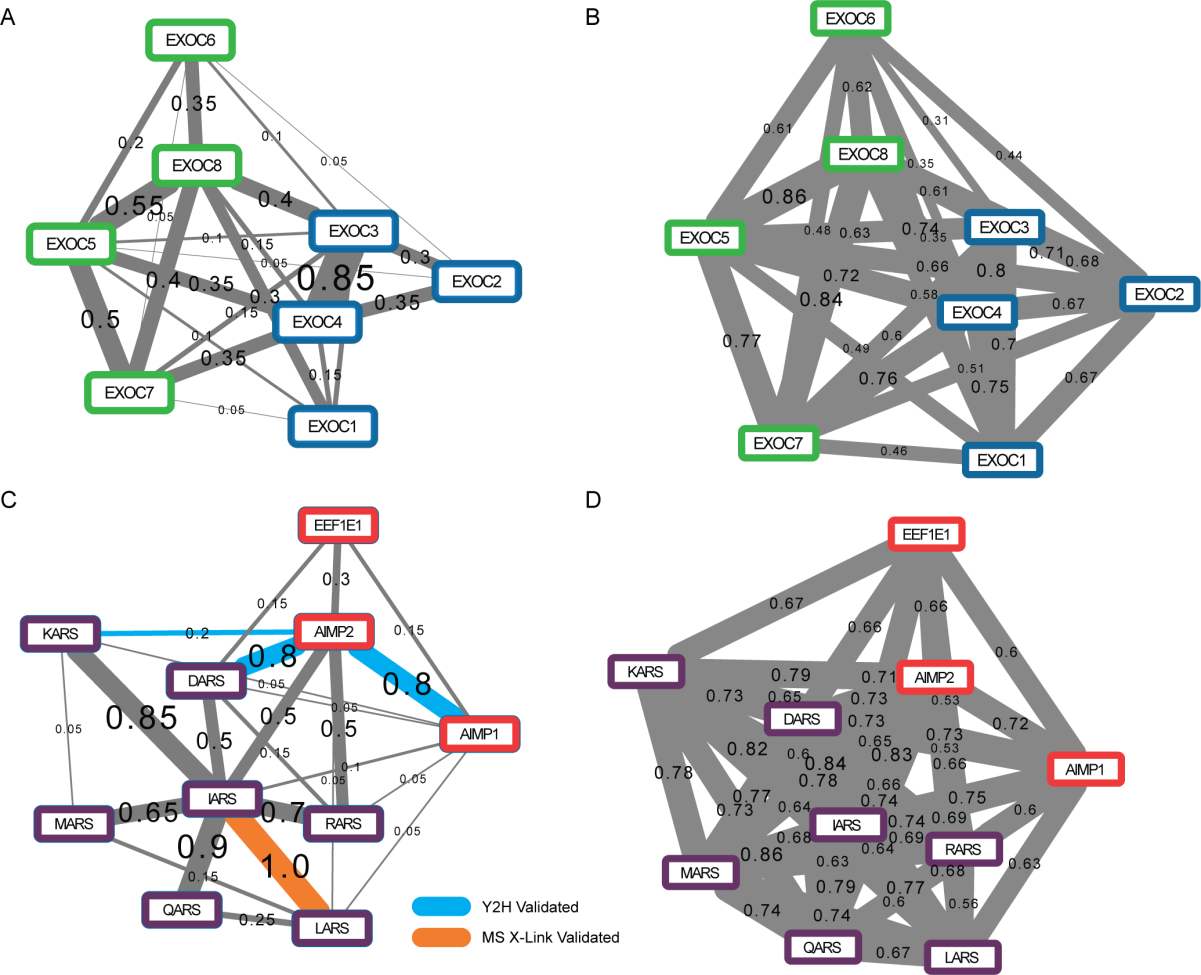
Prediction of direct contacts within human protein complexes of unknown structure. Direct contacts and correlation coefficients were calculated between 8 members of the human exocyst complex (**A**: direct contacts, **B**: correlation) and 10 members of the tRNA multi-synthetase complex (**C**: direct contacts, **D**: correlation). Contact predictions are visualized here by drawing each direct contact prediction or correlation prediction as an edge connecting the relevant subunits. Each predicted direct contact is associated with its prediction score, which indicates the stability support for that interaction. In both complexes, certain direct interactions are strongly supported, suggesting key contacts formed in the three-dimensional organization of these complexes, neither of which has yet been resolved. The comparison between direct contact predictions and correlation predictions indicates that the graphical model removes edges considered conditionally independent from the direct contact network providing high confidence predictions. For **A** and **B**, colors (blue and green) represent known sub-complexes from Heider *et al*. [54]. For **C** and **D**, red represents structural subunits and purple represents synthetases.

The prediction of direct contacts gives an opportunity to characterize the structural architecture of complexes that do not yet have a solved structure. The exocyst complex, for example, is a hetero-octamer involved in tethering vesicles to the plasma membrane and is not well understood at the molecular level [53]. Recent studies by Heider *et al*. [54] and Picco *et al*. [55] have attempted to resolve the yeast exocyst subunit connectivity map using co-purification and nanometer precision fluorescence microscopy, respectively. Interestingly, Heider and colleagues identified two sub-complexes, sub-complex I consisting of Sec3/EXOC1 (denoting yeast/human orthologs), Sec5/EXOC2, Sec6/EXOC3, Sec8/EXOC4 and sub-complex II consisting of Sec15/EXOC6, Sec10/EXOC5, Exo84/EXOC8 and Exo70/EXOC7. Our direct contact predictions, plotted in **Fig 7A**, support the presence of these two sub-complexes in addition to identifying inter-sub-complex contacts between EXOC4—EXOC7, EXOC4—EXOC5, and EXOC3—EXOC8. These contacts along with the highly confident direct contact predicted between EXOC3—EXOC4 (also supported by the Heider *et al*. data) suggests that EXOC3 and EXCO4 form the core subunits of sub-complex I and serve as a bridge to sub-complex II. Likewise, the direct contacts predicted between EXOC5, EXOC7 and EXOC8 suggest they form the core of sub-complex II and are reciprocally responsible for the bridge between sub-complexes. In comparison to the correlation network shown in **Fig 7B** we observe a much denser network with fewer discriminating edges that help to identify the sub-complexes. We also see a range of correlation coefficients that, empirically, have lower precision then their corresponding direct contact probabilities when evaluated on our combined structural benchmark (**Fig 2C**). For instance, the interaction EXOC3-EXOC4 has a direct contact probability of 0.85 which is estimated to have a physical contact precision of ~70% while the corresponding correlation coefficient of 0.8 has an empirical precision of ~45%. This example illustrates the ability of our method to predict high confident physical interactions that discriminate from other indirect interactions.

A second large complex that has thus far eluded structural characterization is the multi-aminoacyl-tRNA synthetase (also known as MARS) complex, which is composed of 9 synthetases and 3 structural subunits (AIMP1/p43, AIMP2/p38, and EEF1E1/p18/AIMP3) and is estimated to be 1 to 1.5 MDa in size [56]. Individual synthetases within the MARS complex are responsible for covalently attaching specific amino acids to their respective tRNAs and are essential for life. However, the function of the conserved supra-molecular assembly remains unclear. Structural studies, although limited, have identified a few trends in terms of overall architecture of the MARS complex [57], including the presence of two sub-complexes mediated by a core AIMP2/p38 subunit. As shown in **Fig 7C**, the direct contact predictions clearly establish AIMP2 as central to the architecture of the MARS complex, and strongly link the two larger structural subunits, AIMP1 with AIMP2. Yeast two-hybrid further supports the AIMP1 and AIMP2 interaction as well as the AIMP2 –DARS interaction and AIMP2 –KARS interaction [8]. Additionally, we see strong interactions between the isoleucyl tRNA synthetase IARS and other members of the complex, including the LARS subunit which is supported by mass spectrometry crosslinking data [11]. This suggests that IARS, in addition to AIMP1 and AIMP2, plays a central role in the physical organization of the MARS complex.

We further compare the direct contact network to the correlation network for the MARS complex (**Fig 7D**). Like the correlation network for the exocyst complex described above, the correlation network for the MARS complex is substantially denser, with many more edges of similar coefficients connecting subunits. Interestingly, we find high correlation edges between subunits DARS and MARS, which do not have an edge in the direct contact network (**Fig 7C**). Since our method attempts to remove spurious conditionally independent edges, this suggests that the correlation coefficient observed between the DARS and MARS subunits can be explained by their mutual interaction with IARS. We see a similar pattern of a high correlation edge absent in the direct contact network including MARS-RARS, MARS-QARS, QARS-RARS, LARS-DARS as well as others. Many of these subunits also interact with the IARS subunit, again suggesting it is the central organizing subunit of the complex. This example demonstrates the utility of direct contact predictions to potentially remove spurious edges from a physical interaction graph.

### Conditional dependency supports a 3D structural model of the human EKC/KEOPS complex

We observed multiple conditionally dependent interactions among a conserved human multi-protein complex with a recently discovered missing subunit. The Endopeptidase-like and Kinase associated to transcribed Chromatin (EKC)/Kinase, Endopeptidase and Other Proteins of small Size (KEOPS) complex is a highly conserved protein complex known to introduce an essential modification to tRNAs across the tree of life [58–60]. The N^6^-threonylcarbamoyladenosine (t^6^A) modification is required for normal cell growth and accurate protein translation in bacteria, archaea, and eukaryotes. While the bacterial and lower eukaryotic components of the EKC/KEOPS complex are known, some of the human subunits are substantially diverged and have only recently been discovered [61, 62].

**Fig 8:**
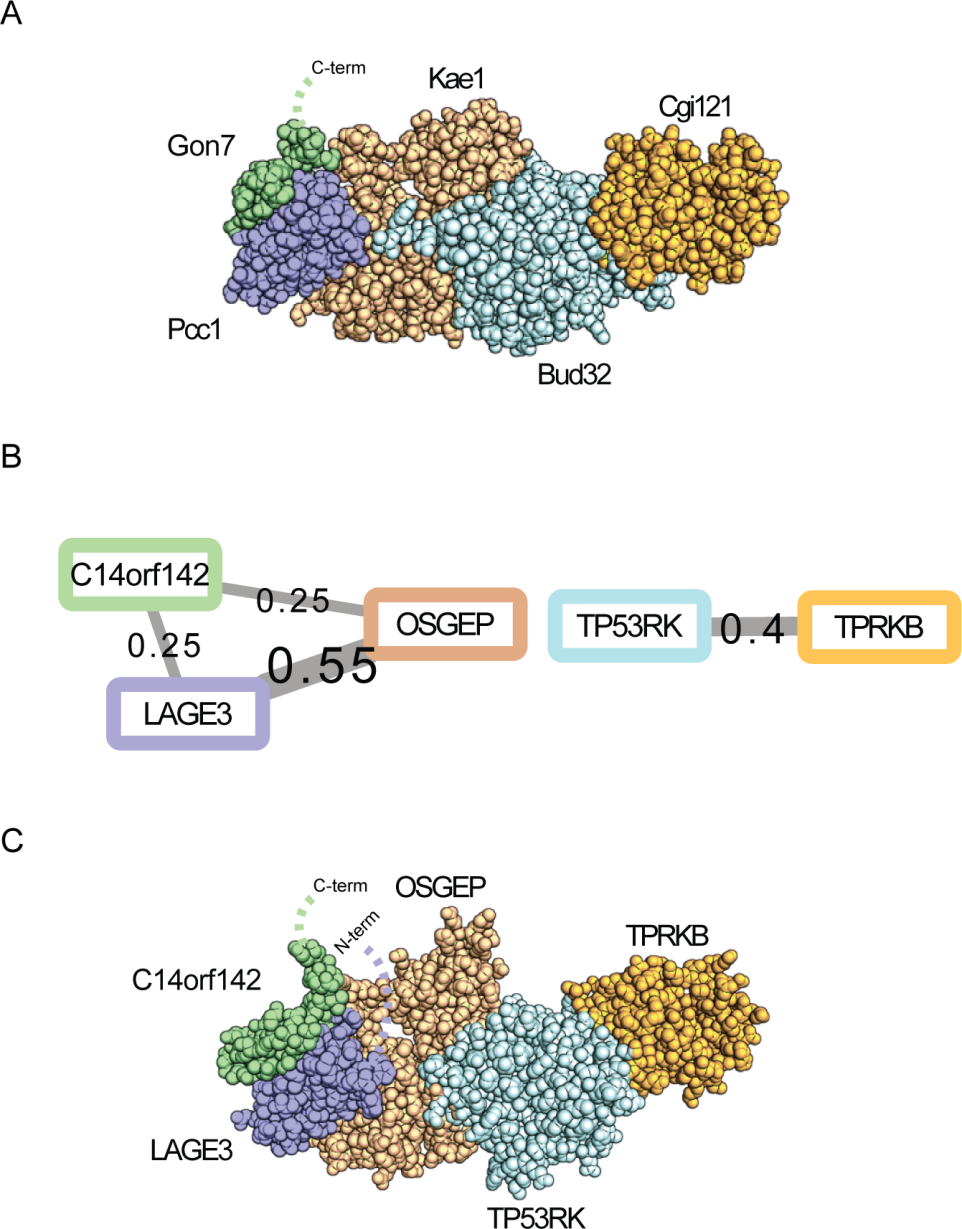
Identification of direct contacts between C14ORF142 and the human EKC/KEOPS tRNA modification complex. **A.** Highly conserved across both prokaryotes and eukaryotes, the EKC/KEOPS complex is thought to consist of 5 proteins, visualized here for the yeast complex based on a combination of modeling and crystal structures (PDB entries 4WX8, 5JMV, 3EN9 and 4WW5). **B.** Conditionally dependent interactions among subunits of the human protein complex highlight specific contacts that match closely to those observed between the yeast proteins, and additionally implicate the human protein C14ORF142 as directly bound to OSGEP and LAGE3. **C.** Using the yeast structure as a homology template, a 3D model was built of the 4 known human subunits and C14ORF142, taking advantage of C14ORF142’s distant similarity to the yeast Gon7 subunit.

In yeast, the complex consists of five proteins, visualized in **Fig 8A**: the atypical TP53 receptor kinase/ATPase (Bud32), the Kinase-Associated Endopeptidase (Kae1), and three small proteins, Cgi121, Pcc1, and Gon7 [58, 60]]. Clear orthologs of four of these occur in humans and have previously been confirmed to participate in the EKC/KEOPS complex: TP53RK (the ortholog of Bud32, known to partially complement a Bud32 mutant [63]), TPRKB (the ortholog of Cgi121), LAGE3 (the ortholog of Pcc1), and OSGEP (the ortholog of Kae1). The yeast Gon7 has generally been thought to be fungi-specific [59, 61], and has no clear mammalian ortholog in major ortholog databases [36, 64].

We found that the conditionally dependent interactions (plotted in **Fig 8B**) strongly supported direct binding of human TP53RK with TPRKB, consistent with expectation from the yeast and archeal crystal structures [40, 41]. Direct binding was also indicated between LAGE3 and OSGEP, again consistent with structural data from archeal homologues [42]. We next observed strong evidence supporting direct binding of OSGEP and LAGE3 with human protein, C14ORF142. Using profile-profile matching, we observe distant but significant homology between C14ORF142 and Gon7 (16% sequence identity and probability score of 92.0), as measured by HHpred [38], which identified Gon7 as the top hit for C14ORF142 from the full non-redundant (reduced to 70% identity) PDB database. This distant sequence similarity strongly supported the observed conditionally dependent protein-protein interactions and suggested that C14ORF142 was indeed likely to substitute for Gon7 within the human complex. Recently, C14ORF142 has been identified as the likely Gon7 ortholog by co-purification with known EKC/KEOPS members [62]. Additionally, the EKC/KEOPS complex was reconstituted *in vitro* and GST-C14ORF142 was shown to bind directly to the OSGEP-LAGE3 sub-complex validating our prediction.

Taking advantage of our predicted direct contacts of C14ORF142 with OSGEP and LAGE3, we constructed a 3D model of the human EKC/KEOPS complex by homology modeling the human proteins onto their yeast orthologs of known 3D structure, including modeling C14ORF142 on the known Gon7 structure (**Fig 8C**). The resulting 3D model accounts for most of the OSGEP, TP53RK, and TPRKB amino acid sequences, but leaves the C-terminal region of C14ORF142 and the N-terminal region of LAGE3 unmodeled, pointing to additional aspects of this complex still yet to be described. Importantly, the model faithfully recapitulates the known functional and interaction data from the literature, the direct contact predictions from the co-fractionation / mass spectrometry datasets, and the newly recognized C14ORF142/Gon7 structural homology, and thus serves to integrate a large body of data into a single model to help guide future mechanistic studies of this ancient human protein complex.

## Conclusion

Knowledge of the three dimensional architecture of a protein complex is highly beneficial to understanding its mechanistic function, but thousands of complexes have thus far proved elusive to traditional structural biology techniques. We present an orthogonal approach in determining aspects of the three dimensional architecture of complexes by analyzing large scale CF-MS datasets. Using our method, we predicted thousands of direct contacts between complex subunits. We expect this resource can be used as a valuable constraint for structurally modeling the many stable protein complexes in the human proteome using available modeling tools [65, 66]. The method should easily extend to new organisms as additional large-scale CF-MS datasets become available. Code and input elution profiles file can be found at https://github.com/marcottelab/direct_contact.

## Supporting information

S1 Table PDB entries of multi-protein complexes in protein contact benchmark. All complexes selected had 1) a PDB entry and 2) > 4 subunits. True = pairs of proteins with >0.0 Å^2^ interface surface area.
(XLS)

S2 Table Full protein contact benchmark. Complete list of contacting and non-contacting protein pairs derived from PDB entries in **S1 Table**.
(XLS)

S3 Table Direct contact predictions. List of direct contact predictions with probability score. Predictions were restricted to only pairs that are co-complex in hu.MAP [32].
(XLS)

S1 File Structural model of human EKC/KEOPS complex. PDB formatted file consisting of the structural homology models of TPRKB, TP53RK, OSGEP, LAGE3 and C14ORF142 interacting in complex.
(PDB)
